# Draft Whole-Genome Sequence of the Black Yeast Aureobasidium pullulans NRRL 62031

**DOI:** 10.1128/mra.00458-22

**Published:** 2023-04-11

**Authors:** Difan Xiao, Lars M. Blank, Till Tiso

**Affiliations:** a Institute of Applied Microbiology, Aachen Biology and Biotechnology, Rheinisch-Westfaelische Technische Hochschule Aachen University, Aachen, Germany; University of California, Riverside

## Abstract

The black-yeast-like *Aureobasidium* is discussed as a versatile cell factory for many biotechnological applications. This article describes the 25.05-Mb draft genome sequence of Aureobasidium pullulans NRRL 62031, which was isolated in Thailand. The genome sequence provides evidence for a plethora of synthesis pathways for valuable secondary metabolites.

## ANNOUNCEMENT

Aureobasidium pullulans is a polyextremotolerant and polymorphic fungus (1, 2). It is ubiquitously distributed in temperate, cold, and arid areas and even in osmotically stressful habitats, which could be attributed to the high degree of metabolic plasticity and adaptation ability of this genus (3, 4). In past decades, different strains of *Aureobasidium* have been reported to produce pullulan, polymalic acid, liamocins, intracellular lipids, siderophores, single-cell proteins, and extracellular enzymes (e.g., amylases, proteases, lipases, cellulases, xylanases, mannanases, and transferases), which are of great interest for industrial, pharmaceutical, environmental, and agricultural applications (1, 2, 5, 6).

A. pullulans strain NRRL 62031 has been confirmed to secrete liamocins (7, 8), a lipase (9), and polymalic acid (10). To prepare DNA for sequencing, yeast extract-peptone-dextrose (YEPD) medium was inoculated using a single colony and incubated at 30°C for 24 h in a shaking cabinet. DNA was isolated with the PureLink genomic DNA (gDNA) kit (Invitrogen, Carlsbad, CA, USA). Briefly, the gDNA was fragmented by acoustic shearing with an S220 instrument (Covaris, Inc., Woburn, MA, USA). The sheared DNA was purified using 0.8× AMPure PB beads (Pacific Biosciences [PacBio], Menlo Park, CA, USA) and end repaired by the addition of NEBNext Ultra II end repair enzyme mix. DNA libraries were validated using a high-sensitivity D1000 ScreenTape on the TapeStation (Agilent Technologies, Palo Alto, CA, USA) and were quantified using a Qubit 2.0 fluorometer (Thermo Fisher Scientific, Carlsbad, CA, USA), as well as real-time PCR (Applied Biosystems, Carlsbad, CA, USA). The libraries were clustered and loaded on an Illumina HiSeq instrument. The qualified libraries were sequenced using a 2 × 150-bp paired-end configuration. The raw sequencing data generated from Illumina HiSeq sequencing were converted into standard FASTQ files and demultiplexed using bcl2fastq v2.17 software. The adapters and low-quality bases with quality scores of <20 were removed using Cutadapt v1.9.1, which yielded 6,471,033,031 bp of clean data. For genome assembly, *k*-mer analysis was performed using Velvet v1.2.10. SSPACE v3.0 was employed to align the sequencing reads to the contigs. The contigs were subsequently assembled into scaffolds depending on the pairwise relationship between the paired-end reads and the size of the inserted segments. GapFiller v1.10 was utilized to align all of the reads from the library to the scaffold sequences. The alignment was used to fill the gaps in the scaffolds, as well as to extend the scaffold sequences to obtain longer sequences with lower rates of undetermined (N) bases (11–15).

The final draft genome assembly comprises 25,051,135 bp, with an average GC content of 50.07%, and consists of 209 scaffolds (*N*_50_, 1,223,087 bp; *L*_50_, 9; sequencing depth, 244.86×). Analysis of biosynthetic gene clusters with antiSMASH v6.0 (16) using default parameters identified several secondary metabolite biosynthetic gene clusters, such as a cluster associated with the production of melanin.

### Data availability.

The whole-genome sequence has been deposited in the NCBI database under BioProject and BioSample accession numbers PRJNA800034 and SAMN25226490, respectively. This whole-genome shotgun project has been deposited in DDBJ/ENA/GenBank under the accession number JALBUZ000000000. The version described in this paper is version JALBUZ010000000. The raw sequencing data have been deposited in the Sequence Read Archive (SRA) under SRA accession number SRR17771678.
